# A Case Report of Non-toxigenic Corynebacterium belfantii With Suspected Diphtheria Infection

**DOI:** 10.7759/cureus.111320

**Published:** 2026-06-22

**Authors:** Kaori Tsumori, Chie Shitada, Tomohiro Ikeda, Minako Sato, Moriyasu Anai, Kaori Mochida, Sunao Ushijima, Mika Inaba, Akane Dobashi, Daihi Sakai, Seiko Fukuda, Kohsuke Kumeda, Chiyomi Sakamoto, Motohide Takahashi, Kuniaki Nagata, Makoto Kuroda

**Affiliations:** 1 Respiratory Medicine, Kumamoto Kenhoku Hospital, Kumamoto, JPN; 2 Toxin and Biologicals Research Laboratory, Kumamoto Health Science University, Kumamoto, JPN; 3 Project Division, The Chemo-Sero-Therapeutic Research Institute (KAKETSUKEN), Kumamoto, JPN; 4 Clinical Laboratory, Kumamoto Kenhoku Hospital, Kumamoto, JPN; 5 Human Resources Department, Corporate Planning and Administration Division, KM Biologics, Kumamoto, JPN; 6 Medical Technology, Faculty of Health Sciences, Kumamoto Health Science University, Kumamoto, JPN

**Keywords:** aspiration pneumonia, average nucleotide identity, corynebacterium belfantii, corynebacterium diphtheriae, infection control, maldi-tof ms, whole-genome sequencing

## Abstract

We report a case of a 69-year-old woman with aspiration pneumonia in whom *Corynebacterium diphtheriae *(*C. diphtheriae*) was initially identified by mass spectrometry but subsequently confirmed as *Corynebacterium belfantii *(*C. belfantii*) through whole-genome analysis. Since sputum culture isolation and subsequent mass spectrometry identification yielded *C. diphtheriae* strain 2511-21 and *Pseudomonas aeruginosa *(*P. aeruginosa*), we notified public health authorities. We implemented isolation measures in accordance with the Infectious Disease Control Law. Diphtheria Toxin production assays, polymerase chain reaction (PCR), and serum antibody tests were all negative, and isolation measures were discontinued following appropriate infection control protocols. Average nucleotide identity (ANI) analysis of the whole-genome sequence revealed that isolate strain 2511-21 showed 99.37% homology with the *C. belfantii* type strain (CIP111412), confirming its identification as *C. belfantii*. Conventional phenotypic identification methods and mass spectrometry cannot reliably differentiate between *C. diphtheriae *and *C. belfantii*, making molecular biological confirmation essential for epidemiologically important bacterial species under infectious disease legislation. Although both *C. belfantii *and *P. aeruginosa* were identified as potentially causative pathogens based on Gram stain and culture findings, the misidentification of *C. belfantii* as *C. diphtheriae *by matrix-assisted laser desorption/ionization time-of-flight mass spectrometry (MALDI-TOF MS) necessitated significant public health measures, highlighting the clinical and regulatory importance of accurate species identification. This case represents the first reported isolation of *C. belfantii *in Japan.

## Introduction

*Corynebacterium diphtheriae *(C. *diphtheriae*) is known as the causative agent of diphtheria and is classified as a category II infectious disease under Japan's Infectious Disease Control Law. When toxigenic* C. diphtheriae* is suspected, hospitals must immediately notify local health authorities to initiate comprehensive public health measures, including contact tracing, epidemiological investigation, and patient isolation, which can create a significant administrative burden. This bacterium is subdivided into four biovars (Gravis, Mitis, Intermedius, and Belfanti) based on biological characteristics. However, in 2018, Dazas et al. proposed reclassifying strains previously designated as biovar Belfanti as an independent new species,* Corynebacterium belfantii *(*C. belfantii*)* *[1].

*C. belfantii* is morphologically and biochemically extremely similar to *C. diphtheriae*, making differentiation using conventional phenotypic identification methods difficult [2]. Mass spectrometry systems widely used in clinical laboratories also display these organisms as *C. diphtheriae mitis*/*belfantii*, failing to distinguish between the two species [3].

Since its proposal as a new species, *C. belfantii* has been reported in only a limited number of cases worldwide, primarily from Europe, and its global prevalence remains unclear [1, 4, 5]. Clinically, *C. belfantii* is generally considered non-toxigenic and is more likely to represent colonization of the upper respiratory tract rather than a true pathogen, although its role in immunocompromised or elderly patients warrants further investigation. Whole-genome sequencing (WGS) and average nucleotide identity (ANI) analysis offer significant advantages over conventional phenotypic methods by enabling definitive species-level identification based on genomic similarity, with a threshold of ≥95% ANI widely accepted for species classification [6].

In this case, although the initial misidentification of the isolate as *C. diphtheriae* by matrix-assisted laser desorption/ionization time-of-flight mass spectrometry (MALDI-TOF MS) triggered a diphtheria-like public health response, including mandatory notification and isolation measures, classical clinical features of diphtheria, such as pseudomembrane formation and *C. belfantii, were* not considered the primary causative pathogen. Nevertheless, its misidentification as *C. diphtheriae* by MALDI-TOF MS necessitated a full public health response in accordance with Japan's Infectious Disease Control Law.

While *C. diphtheriae* requires notification to public health authorities and isolation measures upon isolation under the Infectious Disease Control Law, *C. belfantii *is not subject to these requirements [4, 7, 8]. Therefore, accurate species identification is crucial for infection control purposes. Recent advances have demonstrated the utility of genomic analysis for bacterial species identification [6]. Through this case, we discuss the limitations of conventional identification methods, the importance of appropriate molecular biological identification, and the clinical significance of *C. belfantii*. We also report the genomic information of *C. belfantii *as the first case in Japan.

## Case presentation

A 69-year-old woman was diagnosed with Alzheimer's disease in 2017 and has been residing in a nursing care facility since June 2024. She had a medical history of hypothyroidism and recurrent aspiration pneumonia, for which tube feeding was initiated in June 2025. She was hospitalized for aspiration pneumonia again in September-October 2025. One day before the current admission, she developed increased sputum production and decreased oxygen saturation. On the day of admission, she presented to our emergency department with a fever reaching 38°C.

**Figure 1:**
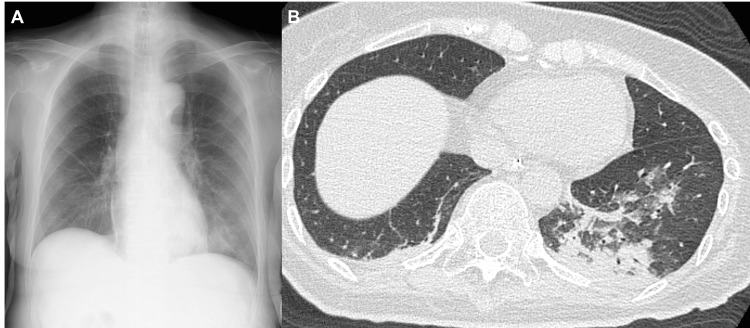
Chest imaging findings on admission (day 1) (A) Chest X-ray Posteroanterior chest radiograph showing consolidation in the left lower lung field. (B) Computed tomography chest CT scan demonstrates consolidation and ground-glass opacities in the left lower lobe. The distribution pattern is consistent with aspiration pneumonia. No pleural effusion or mediastinal lymphadenopathy is observed. These findings led to the diagnosis of aspiration pneumonia and prompted sputum culture, which subsequently yielded the isolate initially suspected as *Corynebacterium diphtheriae*.

On arrival, her vital signs included a body temperature of 38.1°C, oxygen saturation of 93% on room air, and a consciousness level of awake without any stimuli. Physical examination revealed wet rales in the left lower lung field, but neither neck swelling nor oral pseudomembranes were observed. Laboratory studies showed neutrophil-predominant leukocytosis with a white blood cell (WBC) count of 26,570/μL (reference: 3,300-8,600/μL), neutrophils 91.8% (reference: 27-74%), and elevated C-reactive protein of 8.07 mg/dL (reference: 0.00-0.14 mg/dL). Chest radiography and computed tomography demonstrated consolidation and ground-glass opacities in the left lower lobe, leading to a diagnosis of aspiration pneumonia and subsequent hospitalization (Table 1, Figure 1).

**Table 1:** Laboratory findings on admission WBC: white blood cell; Neut: neutrophils; CRP: C-reactive protein; Hb: hemoglobin; PLT: platelets; Alb: albumin; BUN: blood urea nitrogen; Cr: creatinine; Glu: glucose; BNP: brain natriuretic peptide.

Parameter	Result	Reference Range
WBC (×10³/μL)	26.5	3.3–8.6
Neut (%)	91.8	27–74
CRP (mg/dL)	8.07	0.00–0.14
Hb (g/dL)	13.7	11.6–14.8
PLT (×10³/μL)	282	158–348
Alb (g/dL)	3.9	4.1–5.1
BUN (mg/dL)	14	8–20
Cr (mg/dL)	0.42	0.46–0.79
Glu (mg/dL)	224	73–109
BNP (pg/mL)	50.5	0–18.4

**Figure 2:**
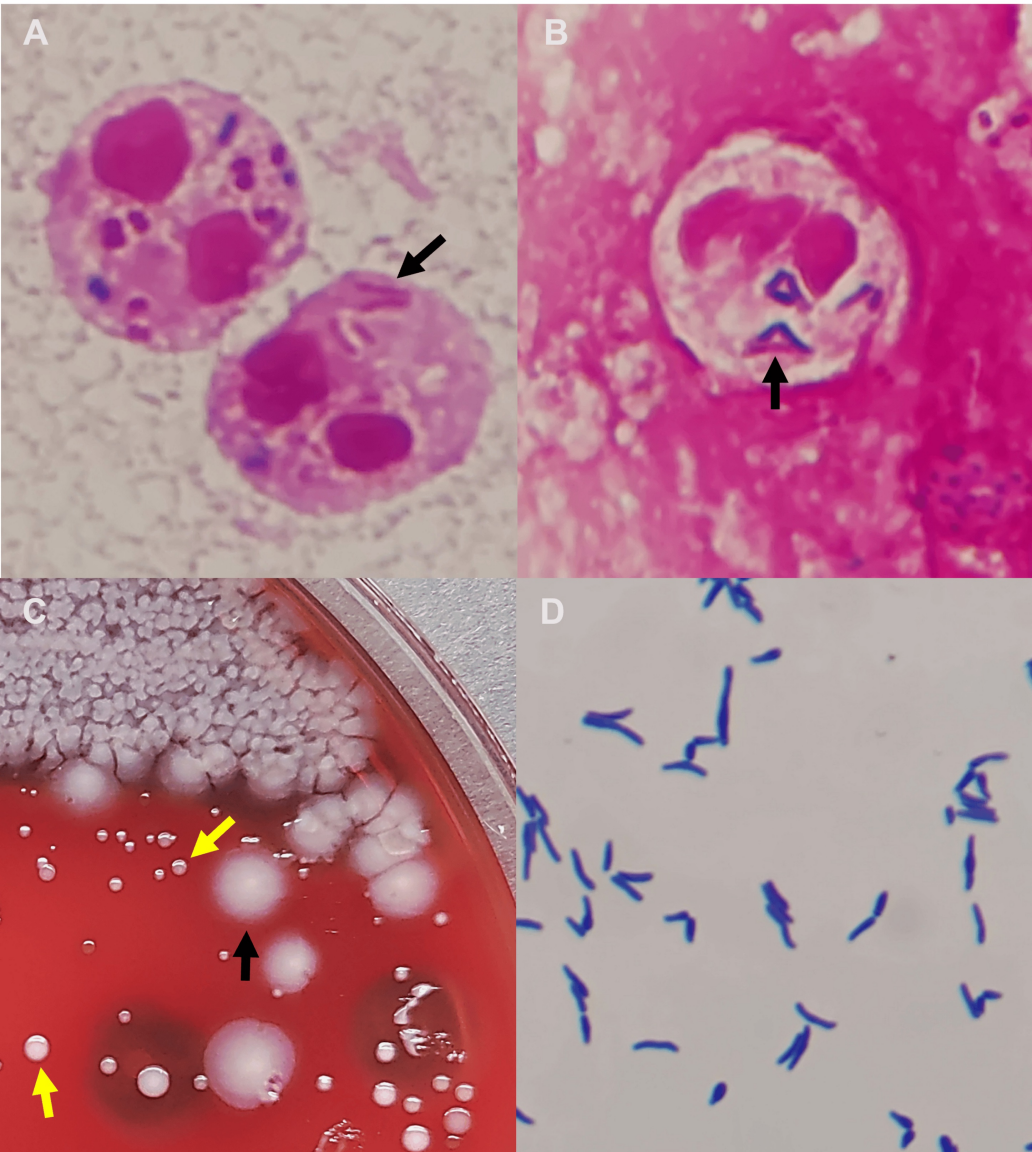
Sputum smear and culture findings (A) Gram-negative rods consistent with *Pseudomonas aeruginosa* within neutrophils (×1000, arrow). (B) Gram-positive rods arranged in V- and Y-shaped configurations, subsequently identified as *Corynebacterium diphtheriae* by Bruker MALDI Biotyper smart (×1000, arrow). (C) Growth on sheep blood agar incubated in 5% CO₂ atmosphere, showing at least two different-sized colonies from the mucopurulent sputum culture. The black arrow indicates a gray-white colony, whereas the yellow arrow indicates a white colony. (D) Gram staining of the white colony indicated by the yellow arrow in Figure 2C, showing characteristic V- and Y-shaped arrangements of gram-positive rods (×1000). The isolate was misidentified as *Corynebacterium diphtheriae* by MALDI-TOF MS, at first. It was correctly identified as *Corynebacterium belfantii* by whole genome sequencing. MALDI-TOF MS: matrix-assisted laser desorption/ionization time-of-flight mass spectrometry.

Gram staining of post-admission sputum revealed both gram-negative and gram-positive rods (Figures 2A, 2B), and culture of mucopurulent sputum on sheep blood agar showed gray-white small colonies and white colonies of varying sizes (Figure 2C). Bruker MALDI Biotyper smart, version 13 (Bruker Daltonics GmbH, Bremen, Germany), a MALDI-TOF MS system, identified* C. diphtheriae *(score 2.31) and *Pseudomonas aeruginosa *(*P. aeruginosa*) (score 2.40). Gram staining of the colony indicated by the yellow arrow in Figure 2C revealed characteristic V- and Y-shaped gram-positive rods (Figure 2D). Based on these identification results, specifically the MALDI-TOF MS identification of *C. diphtheriae* and the characteristic V- and Y-shaped gram-positive rods on Gram staining, notification to public health authorities and isolation measures were implemented on hospital day three in accordance with the Infectious Disease Control Law.

Subsequently, analytical profile index (API) Coryne testing identified the isolate as* C. diphtheriae mitis/belfanti*(90.1%) [9], confirming that conventional phenotypic identification could not differentiate between the two biovars. Antimicrobial susceptibility testing of *C. belfantii* was performed by the broth microdilution method in accordance with the Clinical and Laboratory Standards Institute (CLSI) guidelines (M100-ED33) [10]. The isolate demonstrated susceptibility to most antimicrobial agents tested, including penicillins, cephalosporins, carbapenems, macrolides, clindamycin, minocycline, rifampicin, vancomycin, and teicoplanin. Resistance was observed only to fluoroquinolones [levofloxacin minimum Inhibitory Concentration (MIC) >4 µg/mL, ciprofloxacin MIC >2 µg/mL].

Cell culture method (CCM) using Vero cells [11], polymerase chain reaction (PCR) testing for toxin genes (*tox* gene) [12], and measurement of diphtheria antibodies in patient serum were all negative. On hospital day eight, following confirmation of toxin negativity from public health authorities, isolation measures were discontinued. Antitoxin administration was not performed. Furthermore, Whole-genome sequencing was performed using a hybrid assembly approach combining Illumina short-read sequencing and MinION nanopore long-read sequencing (Oxford Nanopore Technologies, Oxford, UK), as previously described [13]. Average nucleotide identity (ANI) analysis was conducted using FastANI software (National Institutes of Health, Bethesda, USA, and Georgia Institute of Technology, Atlanta, USA). The whole-genome sequence of isolate strain 2511-21 has been deposited in the DNA Data Bank of Japan (DDBJ) database (GenBank accession number: AP045745). ANI analysis revealed 99.37% homology with the *C. belfantii* type strain (CIP111412), and 95.19% homology with the *C. diphtheriae* reference strain (NCTC11397), thereby confirming identification as *C. belfantii*.

Regarding treatment course, ampicillin/sulbactam (ABPC/SBT) was initiated as empirical therapy for nursing and healthcare-associated pneumonia (NHCAP) in accordance with the Japanese Respiratory Society Guidelines for the Management of Pneumonia in Adults 2024 [14], but was changed to piperacillin/tazobactam (PIPC/TAZ) following detection of *P. aeruginosa*.

**Figure 3:**
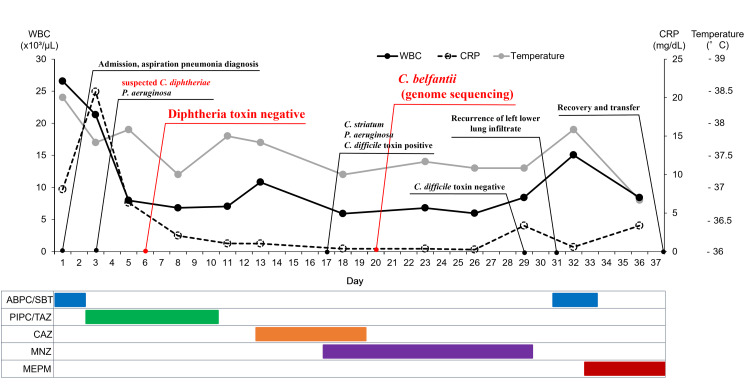
Clinical course Clinical course showing changes in white blood cell count (WBC) and C-reactive protein (CRP) levels along with antimicrobial therapy. The patient was initially treated with ABPC/SBT, which was switched to PIPC/TAZ following the detection of *Pseudomonas aeruginosa*. Although inflammatory markers improved initially, two relapses were observed after discontinuation of antibiotics. CAZ was administered for the first relapse, complicated by* Clostridioides difficile* enteritis treated with MNZ. MEPM was required for the second relapse, after which the patient recovered and was transferred to another hospital. The confirmation of *Corynebacterium belfantii* by whole-genome sequencing, along with negative results by toxin assays, directly informed the decision to discontinue isolation measures and withhold antitoxin administration. ABPC/SBT: ampicillin/sulbactam; PIPC/TAZ: piperacillin/tazobactam; CAZ: ceftazidime; MNZ: metronidazole; MEPM: meropenem.

Initial treatment resulted in improvement of white blood cell count from 26,570/μL to 6,800/μL (reference: 3,300-8,600/μL) and CRP from 8.07 mg/dL to 2.06 mg/dL (reference: 0.00-0.14 mg/dL). However, pneumonia recurred after antibiotic discontinuation, and sputum culture on hospital day 17 yielded *Corynebacterium striatum* and* P. aeruginosa*. Although *C. striatum* has recently gained attention as a causative agent of healthcare-associated infections, we carefully considered the clinical correlation in this case and treated with ceftazidime (CAZ), considering *P. aeruginosa* as the primary pathogen. Concurrent persistent diarrhea with positive stool *Clostridioides difficile* toxin was treated with metronidazole, resulting in improvement. Subsequently, aspiration pneumonia recurred again, ultimately responding to meropenem (MEPM). Antibiotic prescription was based on antimicrobial susceptibility testing results, and the patient recovered and was transferred to another hospital (Figure 3).

## Discussion

*C. belfantii* and *C. diphtheriae* are extremely similar in biochemical characteristics, and API Coryne can only identify both as* C. diphtheriae *mitis/belfanti. Regarding mass spectrometry systems, biovar Belfanti has not been appropriately handled as the new species *C. belfantii*, and current databases lack sufficient spectral data updates for* C. belfantii*, creating a risk of misidentification [3]. Indeed, in this case, Bruker MALDI Biotyper smart identified the isolate as* C. diphtheriae*, which is considered due to an insufficient current database. Since *C. belfantii* was proposed as a new species in 2018 [1], reported cases have been limited worldwide, and the lag in database updates by various manufacturers is considered the primary cause of misidentification [3].

Whole-genome analysis is the most reliable method for differentiating closely related bacterial species. In this case, ANI analysis confirmed the identification of *C. belfantii*, with homology values significantly exceeding the 95% threshold for species classification [5]. The high homology with* C. belfantii* significantly exceeds the 95% threshold for species identification, indicating high reliability of species identification [5]. The genomic information of isolate strain 2511-21 has been registered in international databases as the first report from Japan (accession number: AP045745). This is expected to provide a foundation for future epidemiological analyses and diagnostic improvements.

The final identification of *C. belfantii* through whole-genome sequencing directly influenced clinical management; as *C. belfantii* is not subject to mandatory notification under the Infectious Disease Control Law, isolation measures were discontinued, and antitoxin administration was deemed unnecessary given the confirmed absence of toxin production. In this case, rapid species identification within a short period was possible by implementing CCM, toxin gene PCR, antibody testing, and genomic analysis in parallel.

Albert staining, which is more sensitive than Gram staining for detecting metachromatic granules characteristic of *C. diphtheriae*, was not performed in this case and represents a limitation of our microbiological workup. The diagnosis was ultimately confirmed by whole-genome sequencing and ANI analysis, which is considered the gold standard for species-level identification.

In this case, sputum culture initially detected what was identified as *C. diphtheriae,* but was finally identified as *C. belfantii*. Gram staining of sputum revealed intracellular phagocytosis of both *C. belfantii* and *P. aeruginosa* within neutrophils, suggesting active involvement of both organisms in the infectious process. Quantitative assessment of the initial sputum culture on sheep blood agar demonstrated that *C. belfantii*, forming small white colonies, was present in moderate-to-abundant quantities, occupying approximately half of the culture plate, while *P. aeruginosa*, forming larger colonies, was present in moderate quantities, occupying approximately one-third of the plate. These findings suggest that both organisms may have contributed as causative pathogens, with *C. belfantii* potentially playing a more significant pathogenic role than initially appreciated. Treatment decisions were made based on a comprehensive assessment of Gram stain findings, culture quantities, clinical course, and antimicrobial susceptibility testing results.

Additionally, *C. striatum* detected during the treatment course is a species with increasing reports as a causative agent of healthcare-associated infections [15]. In this case, it was detected during the second recurrence, but clinical correlation was carefully assessed. *Corynebacterium* species, including* C. striatum*, may exhibit multidrug resistance [16], and careful evaluation of their pathogenic role with reference to antimicrobial susceptibility testing results will be necessary in future cases.

In elderly patients with dementia, detection of such rare bacterial species may increase in the future due to changes in oral-pharyngeal flora associated with decreased swallowing function [17]. Healthcare professionals should recognize the limitations of conventional identification methods, including mass spectrometry systems, and actively implement molecular biological confirmation when epidemiologically important bacterial species under infectious disease legislation are suspected.

## Conclusions

Although both *C. belfantii* and *P. aeruginosa* were considered the primary causative pathogens in this case, the clinical and microbiological findings should be interpreted with caution, given the inherent limitations of a single case report. Nevertheless, this case clearly demonstrates that misidentification of *C. belfantii *as *C. diphtheriae *can trigger significant public health consequences, underscoring the importance of molecular confirmation.

This case highlights the critical limitations of current phenotypic identification methods and mass spectrometry in differentiating between *C. diphtheriae* and *C. belfantii*. Accurate species identification through molecular biological methods is essential for appropriate infection control measures, particularly for organisms subject to infectious disease legislation. The genomic data from this first reported case of *C. belfantii* in Japan contribute to improving diagnostic databases and understanding of this emerging pathogen. Healthcare providers must remain vigilant for the potential emergence of rare bacterial species in vulnerable patient populations and ensure access to advanced molecular diagnostic capabilities for epidemiologically significant organisms.
